# *Bacillus subtilis* KCTC 11782BP-Produced Alginate Oligosaccharide Effectively Suppresses Asthma via T-Helper Cell Type 2-Related Cytokines

**DOI:** 10.1371/journal.pone.0117524

**Published:** 2015-02-06

**Authors:** Mi-Ae Bang, Ji-Hye Seo, Joung-Wook Seo, Gyung Hyun Jo, Seoung Ki Jung, Ri Yu, Dae-Hun Park, Sang-Joon Park

**Affiliations:** 1 Food Industry Development Team, Jeonnam Biofood Technology Center, Naju, Korea; 2 Department of Oriental Medicine Materials, Dongshin University, Naju, Korea; 3 Korea Institute of Toxicology, Daejeon, Korea; 4 Research Institute of Bioscience and Biotechnology, Bioresource Inc., Naju, Korea; 5 College of Veterinary Medicine, Kyungpook National University, Daegu, Korea; Mie University Graduate School of Medicine, JAPAN

## Abstract

According to the World Health Organization in 2013, 235 million people are afflicted with asthma. Asthma is a severe pulmonary disease that can be caused by the imbalance of T-helper (Th) type 1 (Th1) and type 2 (Th2) cells, and it is potentially fatal. In this study, we evaluated the anti-asthmatic effect of alginate oligosaccharide (AO), which was prepared from seaweed and converted by *Bacillus subtilis* KCTC 11782BP, in the mouse model of ovalbumin (OVA)-induced asthma. BALB/c mice were divided into the vehicle control (sensitized but not challenged), asthma induction, positive control (1 mg/kg dexamethasone), 50 mg/kg/day AO-treated, 200 mg/kg/day AO-treated, and 400 mg/kg/day AO-treated groups. The numbers or levels of inflammatory cells, eosinophils, and immunoglobulin (Ig) E were measured in bronchoalveolar lavage fluid (BALF), and asthma-related morphological and cytokine changes were analyzed in lung tissues. Our results show that AO dramatically reduced inflammatory cell numbers, eosinophil count, and IgE levels in BALF, and it dose-dependently inhibited asthmatic histopathological changes in the lung. In addition, AO dose-dependently suppressed the expression of CD3^+^ T-cell co-receptors, CD4^+^ Th cells, CD8^+^ cytotoxic T-cell-related factors, macrophages, and MHCII class. AO dose-dependently decreased the expression levels of Th1/2 cells-regulatory transcription factors such as GATA-3 which modulates Th2 cell proliferation and T-bet which does Th1 cell proliferation. The mRNA levels of all Th1/2-related cytokines, except IL-12α, were dose-dependently suppressed by AO treatment. In particular, the mRNA levels of *IL-5*, *IL-6*, and *IL-13* were significantly inhibited by AO treatment. Our findings suggest that AO has the potential to be an anti-asthmatic drug candidate, due to its modulation of Th1/Th2 cytokines, which contribute to the pathogenesis of asthma.

## Introduction

According to the 2013 Asthma Fact Sheet from the World Health Organization, 235 million people are afflicted with asthma [1]. A report in 2010 revealed that approximately 25.7 million patients suffer from asthma in the United States, and children under 17 years old and the elderly are more likely to be affected [2]. Unfortunately, asthma appears to be inappropriately controlled in the United States [3]. There are many inducers of asthma, such as indoor and outdoor allergens, viral infections, and pollution. Pet dander, domestic mites, and cockroaches are indoor allergens, and pollen, mold, and fungi are outdoor allergens. Tobacco smoke, chemical irritants, and air pollution are pollutants [1]. The typical clinical symptoms of asthma include excessive mucus production, goblet cell hyperplasia, epithelial cell shedding, basement membrane thickening, and eosinophil and lymphocyte infiltration. These symptoms eventually lead to airway obstruction [4, 5].

Asthma is a hyperresponsive respiratory disease that is caused by the imbalance of T-helper (Th) cells [4, 5]. Various studies have shown that Th type 1 (Th1)-related cytokines (interleukin [IL]-12 and IFN-γ), Th type 2 (Th2)-related cytokines (IL-4, IL-5, and IL-13), and proinflammatory cytokines (IL-1β, IL-6, and TNF-α) are associated with asthma. Of these, IL-1 is an important mediator of many inflammatory diseases [6], and IL-4 and IL-13 are key regulators of asthma [7]. IL-4 can also mediate the switch from immunoglobulin (Ig) G to IgE and recruit eosinophils [8]. IL-5 regulates the development, activation, migration, and survival of eosinophils and stimulates the expression of IL-6 [9, 10], which is a T- and B-cell growth factor that produces IgE and regulates CD4^+^ T-cell function to induce asthma [9]. IL-12 modulates the balance between the promotion and inhibition of Th1 and Th2 cells, respectively [10, 11]. It also produces IFN-γ [12], which can prevent the switch from IgG to IgE and reduce IgE production [13]. IL-13 is involved in B-cell activation and airway remodeling, which causes excessive mucus production, goblet cell hyperplasia, epithelial cell shedding, basement membrane thickening, and eosinophil and lymphocyte infiltration [14, 15, 16, 17]. Lastly, TNF-α can stimulate granulocyte recruitment and fibroblast proliferation [18].

T-bet is a important transcription factor to control Th1 cell proliferation which could make positive feedback-loop for Th1 cell proliferation through correlated with IFN-γ and/or IL-12 [19, 20] and GATA-3 is the transcription factor to do Th-2-related cytokines such as IL-4 [21].

Bronchodilators, corticosteroids, leukotriene modifiers, theophylline, and anti-IgE therapeutics are currently used for asthma control, although none of these therapies are curative [22]. The common method for controlling asthma is the inhalation of corticosteroids [23]. However, this is associated with several side effects, and it tends to decrease glucocorticoid receptor-binding affinity and T-cell response [24]. Therefore, an increased number of studies have been devoted to finding asthma drug candidates that are obtained from natural products and traditional medicine.

Sodium alginate is a viscous material on the stem of seaweed. It has been used as an anti-inflammatory agent against chronic ulcerative colitis [25] and as an antioxidant [26]. It has also been used in encapsulated materials [27, 28] and microneedles [29]. Alginate oligosaccharide (AO) has been shown to overcome drug resistance and potentiate the action of antibiotics [30], and it prevents salt-induced hypertension in rats [31]. Although the anti-inflammatory and antioxidant properties of AO have been demonstrated in various biological functions, its role in asthma control is unknown. Therefore, we analyzed the effect of *Bacillus subtilis* KCTC 11782BP-produced AO (50, 200, and 400 mg/kg/day for 5 days) on ovalbumin (OVA)-induced asthma in mice.

## Results

### AO dramatically reduced the total number of inflammatory cells and eosinophils, as well as IgE levels, in BALF

**Fig 1:**
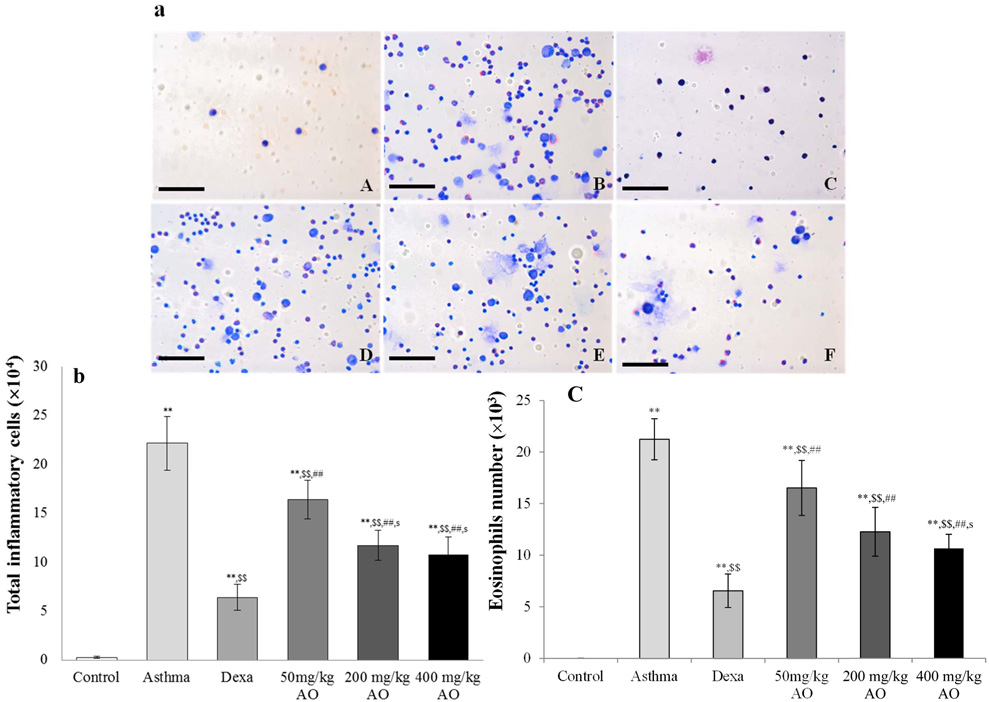
16S rRNA sequences of *Bacillus subtilis* KCTC 11782BP.

**Fig 2:**
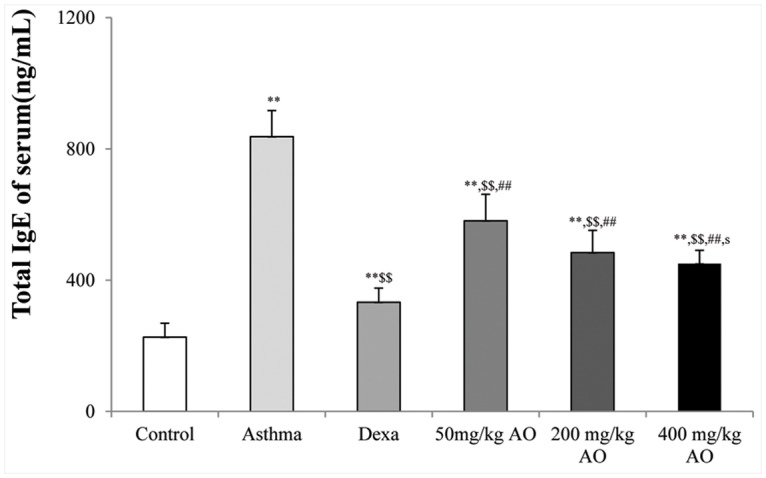
Effects of alginate oligosaccharides (AO) on the ovalbumin (OVA)-induced recruitment of total inflammatory cells and eosinophils in bronchoalveolar lavage fluid (BALF). (a) Total and differential cell counts in BALF were enumerated on slide preparations that were stained with the Kwik-Diff staining set. The numbers of (b) total inflammatory cells and (c) eosinophils in the AO-treated groups were dramatically reduced, compared to the numbers in OVA-challenged, vehicle-treated mice. A, vehicle control; B, asthma induction; C, dexamethasone; D, 50 mg/kg/day AO; E, 200 mg/kg/day AO; F, 400 mg/kg/day AO. Each bar represents the mean ± SEM (n = 6). *p<0.05 vs. control; **p<0.001 vs. control; ^$^ *p*<0.05 vs. asthma induction; ^$$^ *p*<0.001 vs. asthma induction; ^#^ *p*<0.05 vs. dexamethasone; ^##^ *p*<0.001 vs. dexamethasone; ^s^ *p*<0.05 vs. 50 mg/kg/day; ^ss^ *p*<0.001 vs. 50 mg/kg/day; ^p^ *p*<0.05 vs. 200 mg/kg/day; ^pp^ *p*<0.001 vs. 200 mg/kg/day; ^¥^ *p*<0.05 vs. 400 mg/kg/day; ^¥¥^ *p*<0.001 vs. 400 mg/kg/day.Bar = 50 μm.

In the mouse model of OVA-induced asthma, the total number of inflammatory cells (Fig. 1a & 1b) and eosinophils (Fig. 1a & 1c) in BALF was significantly higher than those in vehicle-treated mice (Fig. 1a, 1b & 1c). AO markedly inhibited the OVA-induced expression of inflammatory cells and eosinophils in a dose-dependent manner. Similarly, AO decreased the levels of OVA-induced IgE in a dose-dependent manner (Fig. 2).

### AO dose-dependently reduced asthmatic histopathological changes in the lung

**Fig 3:**
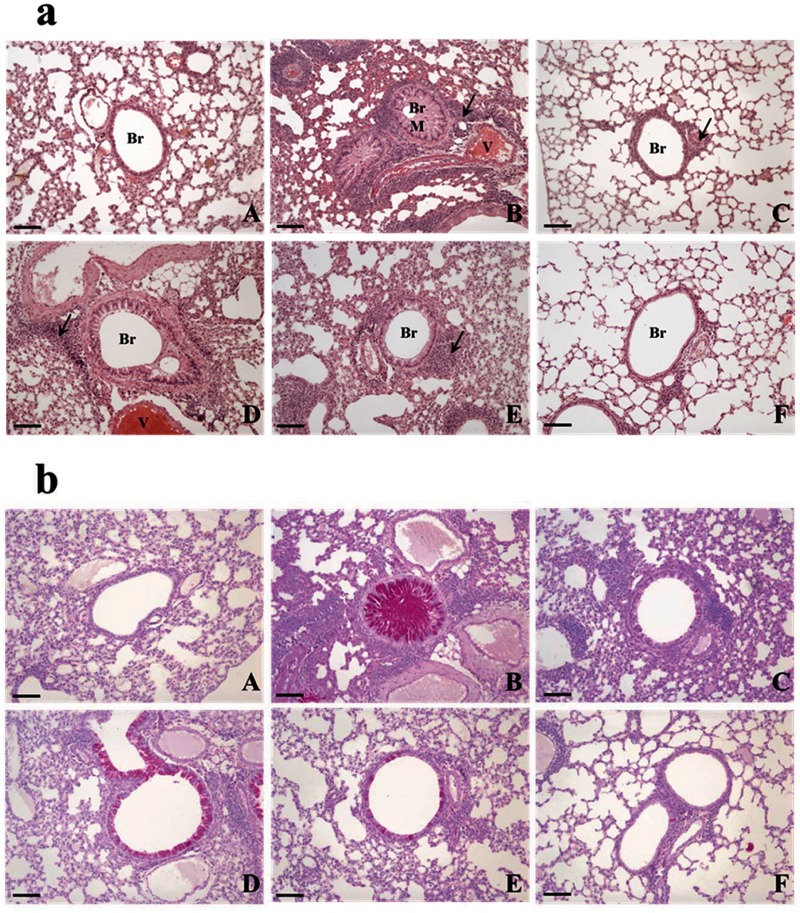
Effects of AO on the OVA-induced upregulation of serum IgE levels. The serum levels of IgE were significantly reduced by AO (*p*<0.01) in a dose-dependent manner, compared with that in OVA-challenged, vehicle-treated mice. A, vehicle control; B, asthma induction; C, dexamethasone; D, 50 mg/kg/day AO; E, 200 mg/kg/day AO; F, 400 mg/kg/day AO. Each bar represents the mean ± SEM (n = 6). *p<0.05 vs. control; **p<0.001 vs. control; ^$^ *p*<0.05 vs. asthma induction; ^$$^ *p*<0.001 vs. asthma induction; ^#^ *p*<0.05 vs. dexamethasone; ^##^ *p*<0.001 vs. dexamethasone; ^s^ *p*<0.05 vs. 50 mg/kg/day; ^ss^ *p*<0.001 vs. 50 mg/kg/day; ^p^ *p*<0.05 vs. 200 mg/kg/day; ^pp^ *p*<0.001 vs. 200 mg/kg/day; ^¥^ *p*<0.05 vs. 400 mg/kg/day; ^¥¥^ *p*<0.001 vs. 400 mg/kg/day.

The morphology of the control lung is shown in Fig. 3aA. OVA induced asthma-related changes in the lung, which included airway remodeling, goblet cell hyperplasia, eosinophil infiltration around the bronchioles and vessels, and mucus plugs (Fig. 3aB). The lungs of mice in the 50 mg/kg/day AO-treated group (Fig. 3aD) exhibited similar histopathological changes to that of the OVA-induced lung (Fig. 3aB), and mucus plugs, goblet cell hyperplasia, and eosinophil infiltration were observed. These morphological changes were also observed in the 200 mg/kg/day AO-treated group, albeit at lower levels than those of the OVA-induced and 50 mg/kg/day AO-treated groups. Interestingly, the morphology of the lungs of mice in the 400 mg/kg/day AO-treated group was similar to that of the control group. In addition, eosinophil infiltration, which was seen in the dexamethasone-treated group, was not observed with 400 mg/kg/day AO treatment.

The mucous glycoprotein was detected using the periodic acid-Schiff (PAS) stain (Fig. 3b). Although glycoprotein secretion was not observed in the control (Fig. 3bA), dexamethasone-treated (Fig. 3bC), and 400 mg/kg/day AO-treated (Fig. 3bF) groups, it was detected in the OVA-induced (Fig. 3bB), 50 mg/kg/day AO-treated (Fig. 3bD), and 200 mg/kg/day AO-treated (Fig. 3bE) groups. Glycoprotein secretion was the highest in the OVA-induced group. Less glycoprotein secretion was observed in the 200 mg/kg/day AO-treated group (Fig. 3bD) compared with that in the 50 mg/kg/day AO-treated group (Fig. 3bE).

### AO suppressed the expression of T cell-related factors, macrophage, and MHC class II in asthma

**Fig 4:**
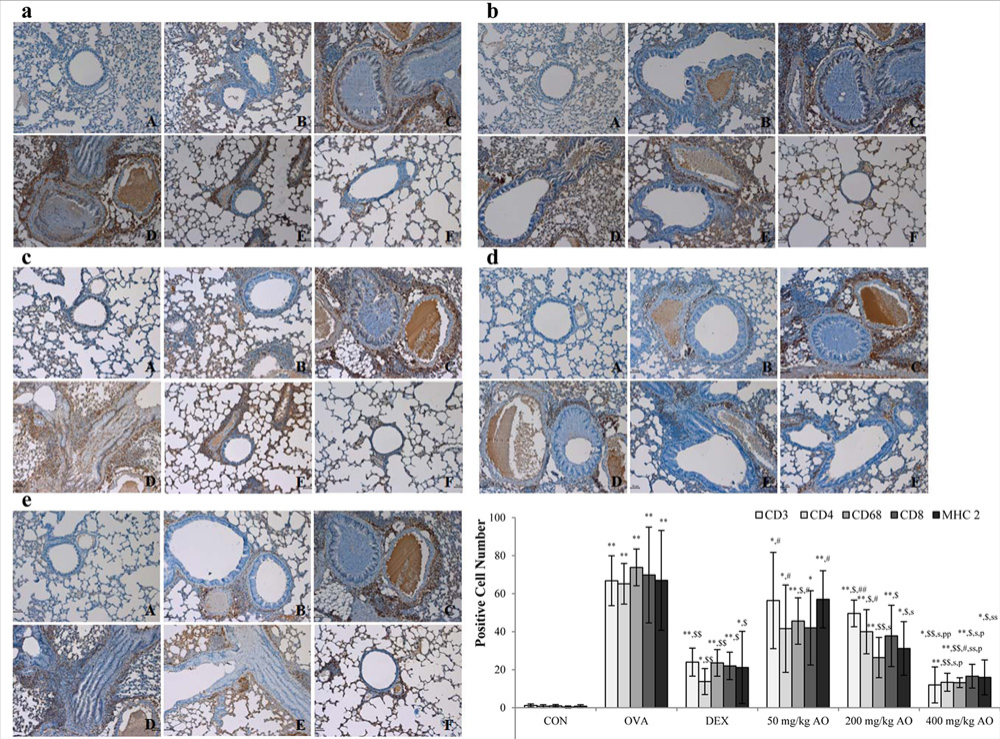
AO dose-dependently suppressed asthma-related histopathological changes in mouse lungs. (a) As observed using the hematoxylin and eosin stain, AO dose-dependently decreased inflammatory cell (eosinophil) infiltration around the vessels and bronchioles, mucus secretion, and goblet cell hyperplasia in the lungs. (b) AO reduced glycoprotein (mucus) secretion in the bronchioles in a dose-dependent manner, as detected by the Periodic acid-Schiff stain. Bar = 10 μm; Arrow = eosinophil infiltration. Br, bronchiole; M, mucus secretion; V, vessel. A, vehicle control; B, asthma induction; C, dexamethasone; D, 50 mg/kg/day AO; E, 200 mg/kg/day AO; F, 400 mg/kg/day AO.

In order to examine the effects of AO on T-cell modulation, changes in the expression of CD3^+^ T-cell co-receptors, CD4^+^ Th cells, and CD8^+^ cytotoxic T cells were measured by immunohistochemistry. AO inhibited the expression of the CD3^+^ T-cell co-receptor (Fig. 4a), CD4^+^ Th cells (Fig. 4b), and CD8^+^ cytotoxic T cells (Fig. 4c). The expression of the CD3^+^ T-cell co-receptor and CD4^+^ Th cells was lower in the 400 mg/kg/day AO-treated group (Fig. 4aF and 4bF) than in the dexamethasone-treated group (Fig. 4aC and Fig. 4bC). AO inhibited the expression of macrophages in a dose-dependent manner (Fig. 4d). In addition, AO dose-dependently suppressed the expression of MHC class II, which is related to react with T helper cell and T cytotoxic cell (Fig. 4e). MHC class II expression was lower in the 400 mg/kg/day AO-treated group (Fig. 4eF) compared with that in the dexamethasone-treated group (Fig. 4eC).

### AO suppressed the expression levels of transcription factors to control Th1 cells proliferation and Th2 cells

**Fig 5:**
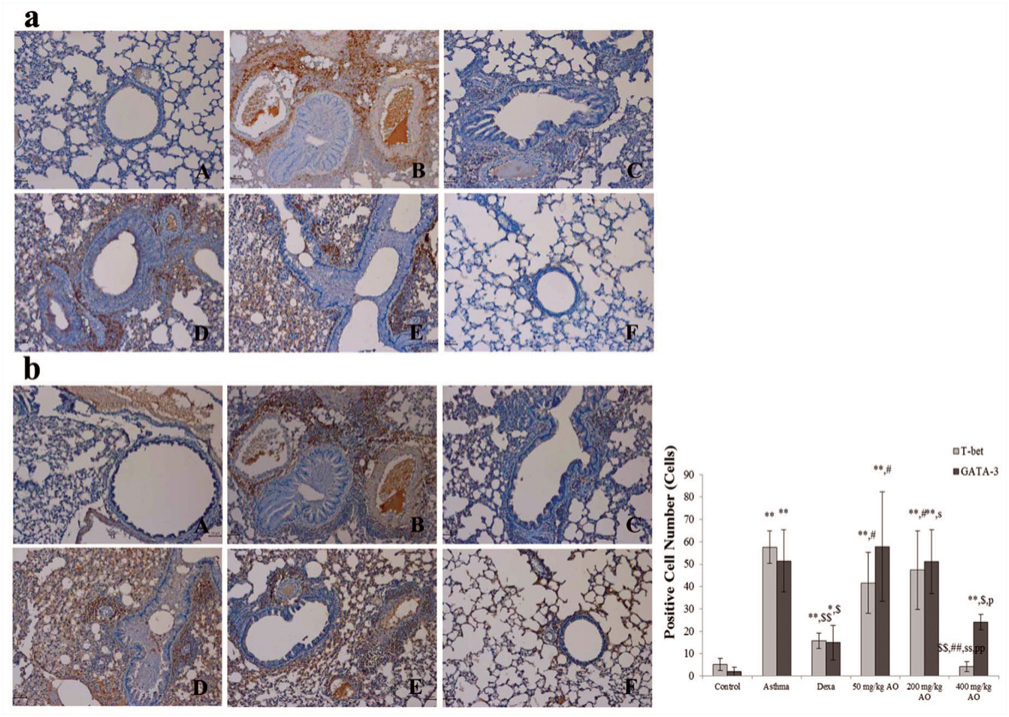
AO dose-dependently suppressed the expression of T-helper (Th) cells, cytotoxic T cells, and the T-cell co-receptor and inhibited the expression of macrophage and MHC class II in asthma. AO (a) inhibited the expression of CD3^+^ T-cell co-receptors in a dose-dependent manner, (b) significantly suppressed the upregulation of CD4^+^ Th cells, (c) downregulated the expression of CD8^+^ cytotoxic T cells, and (d) decreased CD68^+^ macrophasges. (e) AO also inhibited the expression of MHC class II. Immunopositive cells were counted in five randomly selected non-overlapping fields (×200 magnification) of three separately immunostained lung sections per animal. A, vehicle control; B, asthma induction; C, dexamethasone; D, 25 mg/kg/day ACA; E 50 mg/kg/day ACA. CD3: T-cell co-receptor; CD4: Th cell; CD8: cytotoxic T cell; CD68: macrophage; MHC class II: major histocompatibility complex class II molecules. *p<0.05 vs. control; **p<0.001 vs. control; ^$^ *p*<0.05 vs. asthma induction; ^$$^ *p*<0.001 vs. asthma induction; ^#^ *p*<0.05 vs. dexamethasone; ^##^ *p*<0.001 vs. dexamethasone; ^s^ *p*<0.05 vs. 50 mg/kg/day; ^ss^ *p*<0.001 vs. 50 mg/kg/day; ^p^ *p*<0.05 vs. 200 mg/kg/day; ^pp^ *p*<0.001 vs. 200 mg/kg/day; ^¥^ *p*<0.05 vs. 400 mg/kg/day; ^¥¥^ *p*<0.001 vs. 400 mg/kg/day.

The expressions of transcription factor to regulate Th1 cells proliferation (T-bet) and to induce Th2 cells proliferation (GATA-3) were analyzed (Fig. 5). The T-bet expression level was significantly inhibited more by the highest dose (400 mg/kg/day) of AO than by dexamethasone although the level of that was higher in 200 mg/kg/day AO treated group (Fig. 5aE) than in 50 mg/kg/day AO treated group (Fig. 5aD) but there was not the difference of expression between two groups. The expression levels of GATA-3 were observed a similar pattern that AO dose-dependently suppressed the expression levels of GATA-3 (Fig. 5b).

### AO dose-dependently inhibited the mRNA levels of asthma-related cytokines

**Fig 6:**
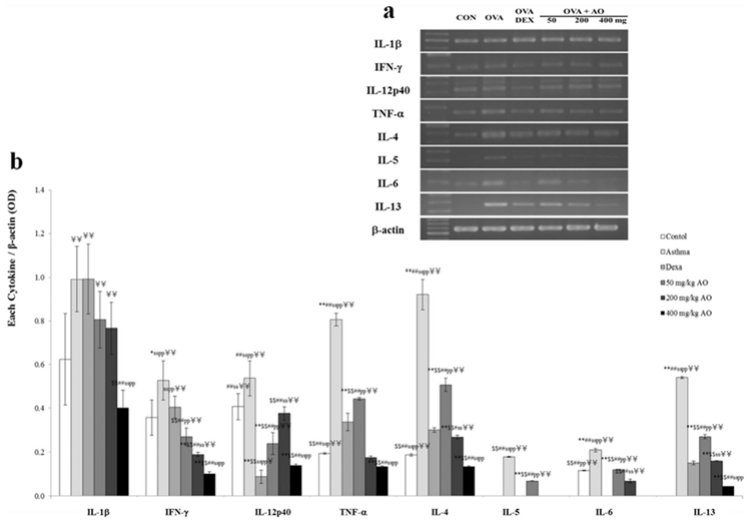
AO reduced the expression of transcription factors to control Th1 cells proliferation and Th2 cells. AO reduced not only the expression of transcription factor, GATA-3 (b), to control Th2 cells proliferation but also the expression of transcription factor, T-bet (a), to do Th1 cells proliferation. Immunopositive cells were counted in five randomly selected non-overlapping fields (×200 magnification) of three separately immunostained lung sections per animal. A, vehicle control; B, asthma induction; C, dexamethasone; D, 50 mg/kg/day AO; E, 200 mg/kg/day AO; F, 400 mg/kg/day. *p<0.05 vs. control; **p<0.001 vs. control; ^$^ *p*<0.05 vs. asthma induction; ^$$^ *p*<0.001 vs. asthma induction; ^#^ *p*<0.05 vs. dexamethasone; ^##^ *p*<0.001 vs. dexamethasone; ^s^ *p*<0.05 vs. 50 mg/kg/day; ^ss^ *p*<0.001 vs. 50 mg/kg/day; ^p^ *p*<0.05 vs. 200 mg/kg/day; ^pp^ *p*<0.001 vs. 200 mg/kg/day; ^¥^ *p*<0.05 vs. 400 mg/kg/day; ^¥¥^ *p*<0.001 vs. 400 mg/kg/day.

The mRNA levels of pro-inflammatory (IL-1β), Th1-related (IL-12α and IFN-γ), and Th2-related (TNF-α, IL-4, IL-5, IL-6, and IL-13) cytokines were measured (Fig. 6a) and analyzed by ImageJ software (Fig. 6b). Only the highest dose (400 mg/kg/day) of AO reduced *IL-1β* mRNA levels. With the exception of *IL-12α*, the mRNA levels of the other Th1/2 cell-related cytokines were dose-dependently suppressed by AO treatment, and *IL-5*, *IL-6*, and *IL-13* mRNA levels were significantly inhibited by AO treatment. The mRNA level of *IL-12α* in the 400 mg/kg/day AO-treated group was slightly increased compared to that in the control group; however, this was not statistically significant. Furthermore, the mRNA levels of *IL-12α* in the dexamethasone-treated and 400 mg/kg/day AO-treated groups decreased in the same pattern. In contrast, treatments with lower doses of AO restored *IL-12α* mRNA levels.

### AO reduced the expression of Th2- and Th1-related cytokines

**Fig 7:**
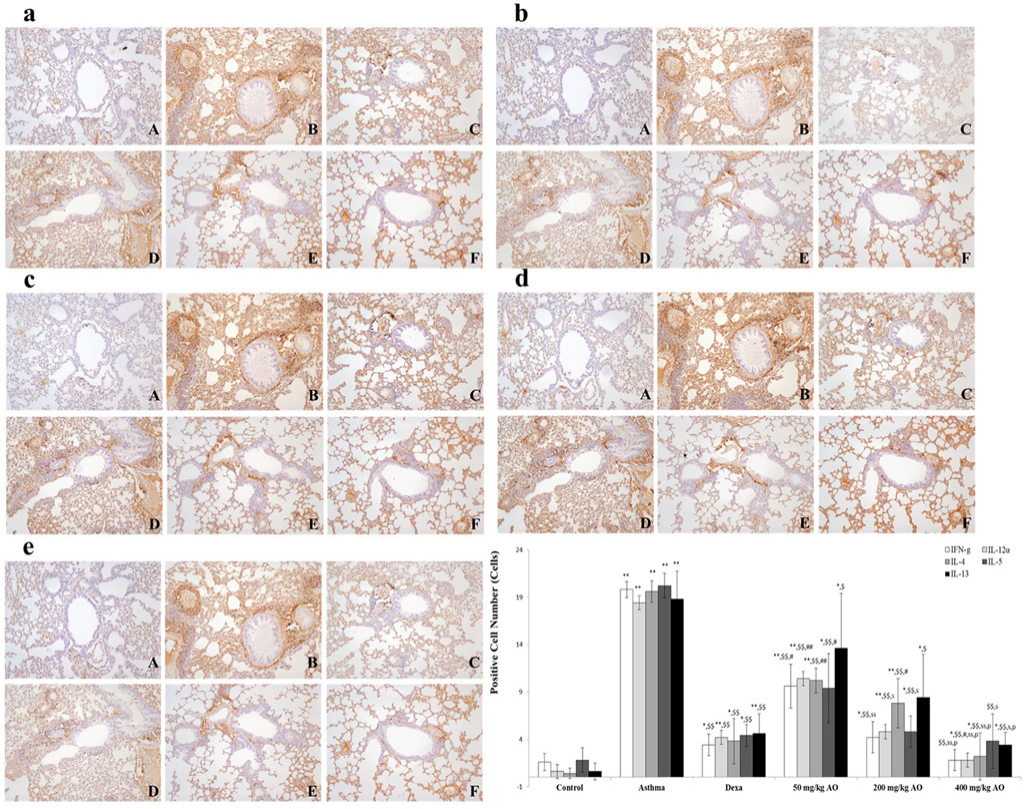
AO suppressed the expression of Th1/2-related cytokines in OVA-induced asthma. Treatment with 400 mg/kg/day AO for 5 days suppressed the expression of *IL-1β* mRNA. AO also decreased the mRNA levels of Th2-related cytokines (TNF-α, IL-4, IL-5, IL-6, and IL-13) and Th1-related cytokines (IFN-) via dose-dependent manners, and it significantly inhibited the expression of *IL-5*, *IL-6*, and *IL-13* mRNA. AO slightly increased the mRNA level of *IL-12α* compared to that of control, and treatment with dexamethasone inhibited *IL-12α* mRNA expression, which was recovered by treatment with 40 mg/kg/day or 200 mg/kg/day AO. At 400 mg/kg/day, AO down-regulated *IL-12α* mRNA expression. *p<0.05 vs. control; **p<0.001 vs. control; ^$^ *p*<0.05 vs. asthma induction; ^$$^ *p*<0.001 vs. asthma induction; ^#^ *p*<0.05 vs. dexamethasone; ^##^ *p*<0.001 vs. dexamethasone; ^s^ *p*<0.05 vs. 50 mg/kg/day; ^ss^ *p*<0.001 vs. 50 mg/kg/day; ^p^ *p*<0.05 vs. 200 mg/kg/day; ^pp^ *p*<0.001 vs. 200 mg/kg/day

Asthma is characterized by the increased secretion [32] and imbalance of Th2- and Th1-derived proinflammatory cytokines [4, 5]. We evaluated the localization and expression of Th1-related cytokines (IFN-γ and IL-12) and Th2-related cytokines (IL-4, IL-5, and IL-13), which were all in their secreted forms. Cytokine expression was investigated near the bronchioles and pulmonary vessels. In the OVA-induced lung, both Th1-related and Th2-related cytokines were overexpressed. AO suppressed not only Th2-related cytokines, such as IL-4 (Fig. 7c), IL-5 (Fig. 7d), and IL-13 (Fig. 7e), but also Th1-related cytokines, such as IFN- γ (Fig. 7a) and IL-12 (Fig. 7b).

## Discussion

Asthma is progressed by exposure of various allegic antigens, and the severity of the disease is related with infiltrated inflammatory cells and its cytokine expression. Untill now it has known that Th2-cytokines relevant to CD4-T cells, eosinophil and macrophages play a major role in the development of asthma.

In this study, we demonstrate that AO downregulated the number of total inflammatory cells and eosinophils, as well as IgE levels, in BALF. AO treatment also inhibited OVA-induced histopathological changes, such as excessive mucus production, goblet cell hyperplasia, epithelial cell shedding, basement membrane thickening, and eosinophil infiltration. Furthermore, compared with dexamethasone, AO significantly inhibited the expression of the CD3^+^ T-cell co-receptor, CD4^+^ T helper cell, CD68^+^ macrophage, and MHC class II. With the exception of IL-12, the mRNA levels of asthma-related cytokines were suppressed, and the mRNA expression of *IL-4* and *IL-5* was almost completely inhibited. Finally, AO downregulated not only the expression levels of transcription factors to control Th1/2 cell proliferation, such as T-bet and GATA-3 but also the expression levels of Th1/2 cytokines, such as IFN-γ, IL-12, IL-4, IL-5, and IL-13.

Yoshida T et al. (2004) reported that alginic acid oligosaccharide had suppressed IL-4 induction and had promoted IL-12 increasing [33]. Asthma can occur via a serial effect of various cytokines; however, it cannot be a result of one specific cytokine or factor [21]. Allergen-related asthma stimulates dendritic T cells, and the release of IL-5, IL-4, and IL-13 is increased. IL-5 regulates eosinophil function and stimulates IL-6 expression [9, 10], IL-4 switches IgE and recruits eosinophils [8], and IL-13 stimulates B cells and airway remodeling to cause bronchoconstriction [13, 14, 15, 16]. Other Th2-related cytokines, such as IL-12 and IFN-γ, and Th1-related cytokines play roles in suppressing the occurrence of asthma. IL-12 promotes the function of Th1 cells, the differentiation of Th2 cells, and the production of IgE [18]. MHC class II molecules are usually found on antigen-presenting cells such as dentritic cells, macrophages, some endothelial cells, and B cells. It has important role to present extracellular molecules to immune cells [34].

Th2-related cytokines (IL-5, IL-6, and IL-13) can contribute to the induction of asthma [9, 10, 13, 14, 15, 16]. In this study, the mRNA level of *IL-13* was decreased by AO via a dose-dependent manner, and *IL-5* and *IL-6* mRNA levels were completely blocked by treatment with 400 mg/kg/day AO. Furthermore, AO dose-dependently suppressed the expression of IL-5 and IL-13. Although treatments with dexamethasone or 400 mg/kg/day AO decreased the mRNA level of *IL-12α*, lower doses of AO increased *IL-12α* mRNA levels. The roles of IL-12 in asthma occurrence are well known. The same cannot be said about IFN-γ, which exhibits contrasting effects on the occurrence of asthma. For example, hydrocortisone is an anti-asthmatic drug that decreases IFN-γ expression, but the *Caenorhabditis elegans* extract can ameliorate asthma by increasing IFN-γ expression [26]. Several studies have reported that BALF IFN-γ levels are elevated in asthmatic patients [27, 28], and methacholine-induced airway hyperresponsiveness is more severe in IFN-γ transgenic mice than in normal mice [29]. Although alginic acid oligosaccharide had inhibited IL-4 induction and had stimulated IL-12 induction [33], in this study, we found that AO decreased IFN-γ levels and the expression of other Th1-related and Th2-related cytokines in OVA-induced mice.

Although steroids have been used as anti-asthmatic drugs, their associated side effects have forced investigators to look for more efficacious and less adverse drug candidates. Because various cytokines can contribute to the pathogenesis of asthma, compounds that actively modulate these cytokines can have potential therapeutic benefits. Results from this study suggest that AO may show promise as an anti-asthmatic drug candidate, due to its ability to suppress various types of cytokines.

## Materials and Methods

### AO production and confirmation

AO was produced from sodium alginate, which was extracted from the brown seaweed *Laminaria hyperborean*. *Bacillus subtilis* KCTC 11782BP [35] was seed-cultured in 150 mL culture medium, which contained 0.1% sodium alginate, 1% peptone, 1% yeast extract, 0.2% NaCl, 0.2% K_2_HPO_4_, 0.1% KH_2_PO_4_, and 0.1% MgSO_4_, at 37°C for 3 days with shaking (150 rpm). It was then added to the alginate medium (5 L), which contained 3% sodium alginate, 1% peptone, 1% yeast extract, 0.2% NaCl, 0.2% K_2_HPO_4_, 0.1% KH_2_PO_4_, 0.1% MgSO_4_, 0.05% CaCl_2_, and 0.1% trace element (pH 7.0). After incubating at 37°C for 14 days, the culture supernatant was obtained by centrifugation (10,000 g, 30 min). AO, which was 10 kDa below the molecular weight, was separated using the Amicon filter (Millipore, Billerica, MA, USA) and then freeze dried.

After the separation by molecular weight below 10 kDa, *Bacillus subtilis* KCTC 11872BP-produced AO was confirmed by the YL9100 high-performance liquid chromatography system (YL Instrument, Anyang, Kyounggi, Korea), and the mannuronate oligosaccharides DP5 (ELICITYL SA, Crolles, France) was used as the standard (S1 Fig.).

### *Bacillus subtilis* KCTC 11782BP

**Fig 8:**
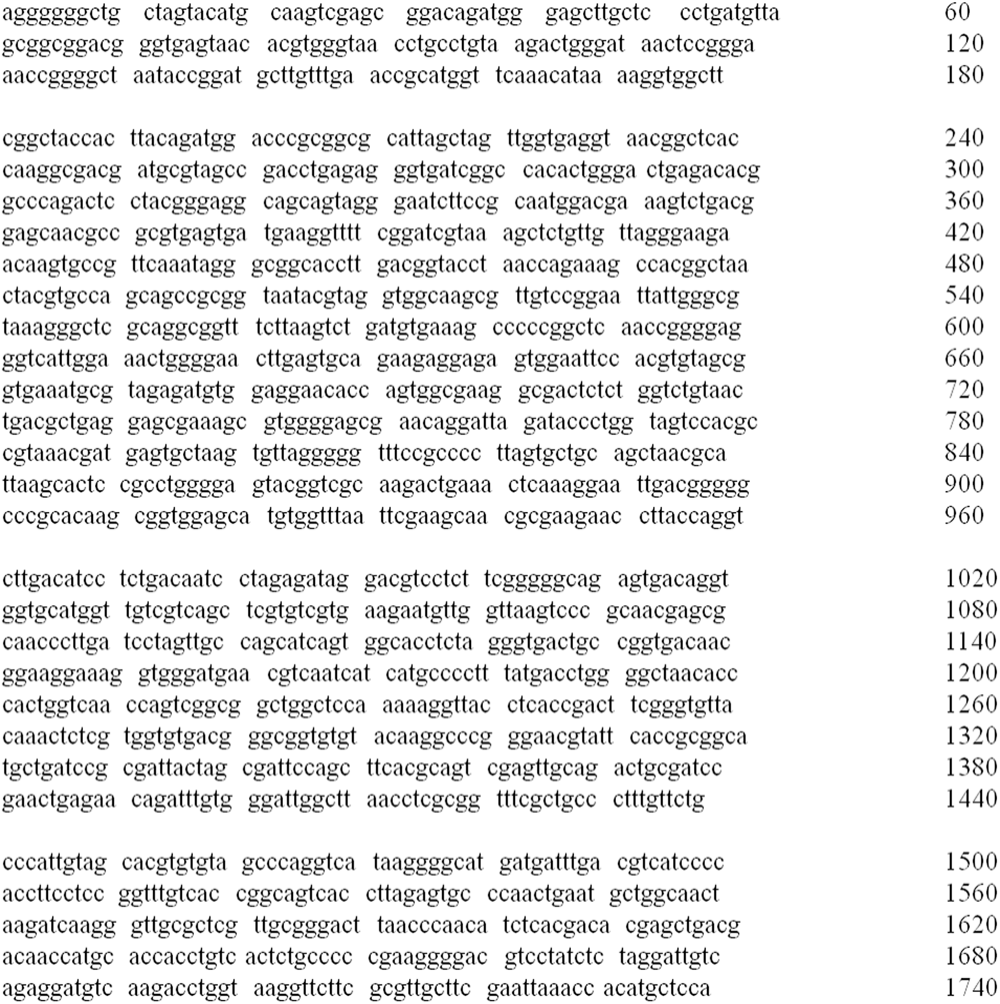
AO reduced the expression of Th1- and Th2-related cytokines. OVA induced the expression of Th1- and Th2-related cytokines. AO reduced the expression of Th1-related cytokines, such as (a) IFN-γ and (b) IL-12α, and Th2-related cytokines, such as (c) IL-4, (d) IL-5, and (e) IL-13, in the lungs. Immunopositive cells were counted in five randomly selected non-overlapping fields (×200 magnification) of three separately immunostained lung sections per animal. A, vehicle control; B, asthma induction; C, dexamethasone; D, 50 mg/kg/day AO; E, 200 mg/kg/day AO; F, 400 mg/kg/day. *p<0.05 vs. control; **p<0.001 vs. control; ^$^ *p*<0.05 vs. asthma induction; ^$$^ *p*<0.001 vs. asthma induction; ^#^ *p*<0.05 vs. dexamethasone; ^##^ *p*<0.001 vs. dexamethasone; ^s^ *p*<0.05 vs. 50 mg/kg/day; ^ss^ *p*<0.001 vs. 50 mg/kg/day; ^p^ *p*<0.05 vs. 200 mg/kg/day; ^pp^ *p*<0.001 vs. 200 mg/kg/day; ^¥^ *p*<0.05 vs. 400 mg/kg/day; ^¥¥^ *p*<0.001 vs. 400 mg/kg/day.

The strain was collected from the South Sea water in Korea (126:47E, 34:18N) and identified using alginate medium. The isolated microorganisms were accurately identified via phylogenetic and sequence comparison analyses of the 16S rRNA sequences (Fig. 8). These sequences were 98% consistent with those of *Bacillus subtilis* LL3. The identified strain was deposited in the Korean Collection for Type Cultures.

### Animal experiments

Using the same methods, two animal studies which were performed at different times were conducted. Eighty-four female BALB/c mice were purchased from Orient Bio Inc. (Seungnam, Korea) and divided into six groups according to treatment: (1) vehicle control (sterilized tap water), (2) OVA-induced asthma model, (3) 1 mg/kg/day dexamethasone with OVA induction, (4) 50 mg/kg/day AO with OVA induction, (5) 200 mg/kg/day AO with OVA induction, and (6) 400 mg/kg/day AO with OVA induction. On days 1 and 8, all mice except those used as the vehicle control were sensitized via intraperitoneal injections of 20 μg OVA (Sigma Chemical Co.) and 1 mg aluminum hydroxide hydrate (Sigma Chemical Co.) in 500 μL saline. From day 21 to day 25, the mice were challenged once daily with 5% OVA for 30 min using a nebulizer (3 mL/min, NE-U17, OMRON Co. Ltd., Kyoto, Japan). During the same 5-day period, the treatment groups were also treated once daily with oral doses of sterilized tap water, dexamethasone, 50 mg/kg/day AO, 200 mg/kg/day AO, or 400 mg/kg/day AO at 1 h prior to OVA challenge. The mice in the vehicle control group were sensitized with OVA according to the same procedures as the other groups of mice (20 μg OVA and 1 mg aluminum hydroxide hydrate in 500 μL saline), after which they were exposed to saline and aluminum hydroxide hydrate by nebulizer for 5 consecutive days.

### Ethic statement

All experiments were approved by the Institutional Animal Care and Use Committee at Kyungpook National University (Approval No. 1003–0028 & 1003–0028[1]).

### BALF analysis

One day after the final treatment, the mice were anesthetized with intraperitoneal injections of pentobarbital (60 mg/kg), and the tracheas were cannulated with disposable animal feeding needles. Lavages were performed with three 0.4-ml aliquots of cold phosphate-buffered saline (PBS). The BALF samples were collected and immediately centrifuged. The cell pellets were resuspended in PBS for total and differential cell counts. The number of total cells was counted using a hemocytometer, and the number of eosinophils in BALF was counted on cytospin preparations that were stained with the Kwik-Diff staining set (Thermo Fisher Scientific Inc., Pittsburgh, PA, USA). The levels of IgE in the serum were measured using a specific mouse IgE enzyme-linked immunosorbent assay kit (Shibayagi Co., Ltd), according to the manufacturer’s protocols.

### Histopathological analysis

Lung tissues were fixed in 10% (v/v) formaldehyde solution, dehydrated in a graded ethanol series (99.9%, 90%, 80%, and 70%), and embedded in paraffin. Paraffin-embedded lung tissues were then sectioned (4 μm) longitudinally and stained with hematoxylin and eosin. The sections were also stained with PAS for the semi-quantitative analysis of glycoproteins.

### Reverse-transcription polymerase chain reaction (RT-PCR)

To evaluate proinflammatory cytokine expression, total RNA was extracted from lung tissues with the RNeasy Mini Kit (QIAGEN, Frederick, MD), according to the manufacturer’s instructions. Total RNA (100 ng) was used as template for the reverse transcription reaction. Primers were synthesized for the semi-quantitative PCR as follows: IL-1 forward, 5′-CTCAGAAGCAGAGCACAAGC-3′, and reverse, 5′-CTCAGTGCAGGCTATGACCA-3′; IFN-γ forward, 5′-AATGAACGCTACACACTGCA-3′, and reverse, 5′-TGAAGAAGGTAGTAATCAGG-3′; IL-12α forward, 5′-GCCAGGTGTCTTAGCCAGTC-3′, and reverse, 5′-ATGGCCTGGAACTCTGTCTG-3′; TNF-α forward, 5′-CCACATCTCCCTCCAGAAAA-3′, and reverse, 5′-AGGGTCTGGGCCATAGAACT-3′; IL-4 forward, 5′-CCAGCTAGTTGTCATCCTGC-3′, and reverse, 5′-GTFATGTGGACTTGGACTCA-3′; IL-6 forward, 5′-TTGCCTTCTTGGGACTGATG-3′, and reverse, 5′-CAGAATTGCCATTGCACAACT-3′; IL-12α forward, 5′-GCCAGGTGTCTTAGCCAGTC-3′, and reverse, 5′-ATGGCCTGGAACTCTGTCTG-3′; IL-13 forward, 5′-TCTGTGTAGCCCTGGATTCCC-3′, and reverse, 5′-CCGTGGCGAAACAGTTGCTT-3′; β-actin forward, 5′-GAAATCGTGCGTGACATC-3′ and reverse, 5′-GCTTGCTGATCCACATCT-3′. The PCR cycles consisted of denaturation at 94°C for 30 s, annealing at 58°C for 30 s, and extension at 72°C for 60 s for 35 cycles. PCR products were separated by electrophoresis through a 2% agarose gel, stained with ethidium bromide, and then detected using ultraviolet light. For the semi-quantitative analysis of PCR bands, the density of each band was measured with a computer imaging device and accompanying software (Bio-Rad, Hercules, CA). The acquired images were analyzed with ImageJ software [36].

### Immunohistochemical analysis

Deparaffinized tissue sections were treated with 3% hydrogen peroxide in methanol for 10 min to remove endogenous peroxidase. Antigen retrieval was carried out with sodium citrate buffer (0.1 M) using the microwave method. The slides were incubated with normal serum to block nonspecific binding and then incubated overnight at 4C with primary antibodies (diluted 1:100–1:200) such as rabbit anti-mouse CD3 polyclonal antibody (abcam, ab5690), rat anti-mouse CD4 monoclonal antibody (eBioscience, 14–9766), rat anti-mouse CD8 monoclonal antibody (Santa Cruz, sc18913), rabbit anti-mouse CD68 polyclonal antibody (Bioss, bs-0649R), rat anti-mouse MHC class II monoclonal antibody(Santa Cruz, sc-59318), rabbit anti-mouse Tbx21/T-bet polyclonal antibody (Bioss, bs-3599R), goat anti-mouse GATA-3 antibody (ORIGENE, TA305795), rat anti-mouse IFN- monoclonal antibody (Santa Cruz, sc-74104), rat anti-mouse IL-12p40 monoclonal antibody (Santa Cruz, sc-57258), rat anti-mouse IL-4 monoclonal antibody (Santa Cruz, sc-73318), rabbit anti-mouse IL-5 polyclonal antibody (Santa Cruz, sc-7887), or goat anti-mouse IL-13 polyclonal antibody (Santa Cruz, sc-1776). The slides were incubated for 2 h with biotinylated secondary antibody (1:500; DAKO, Carpinteria, CA) and with horseradish-peroxidase conjugated streptavidin. Signals were detected using the 3,3-diaminobenzidine tetrahydrochloride substrate chromogen solution, and the cells were counterstained with Mayer’s hematoxylin. To determine the number of positively stained cells, we counted cells in five randomly selected non-overlapping fields (×200 magnification) of three separately immunostained lung sections per animal (n = 7 per group).

### Statistical analysis

Results are expressed as mean ± standard deviation (SD). Group differences were evaluated by one-way analysis of variance, followed by Dunnett’s multiple comparison test. Significance was considered at *p*<0.01 or *p*<0.05.

## Supporting Information

S1 Fig High-performance liquid chromatography analysis of *Bacillus subtilis* KCTC 11782BP-produced alginate oligosaccharide (AO). (a) The peak of mannuronate oligosaccharides DP5 was increased for about 1 min after the standard was injected, from the 4-min mark to the 5-min mark. (b) The peak of *Bacillus subtilis* KCTC 11782BP-produced AO was increased for about 1.5 min after the standard was injected, from the 4-min mark to the 5.5-min mark.
(TIF)
