# Correction: *Bacillus subtilis* KCTC 11782BP-Produced Alginate Oligosaccharide Effectively Suppresses Asthma via T-Helper Cell Type 2-Related Cytokines

**DOI:** 10.1371/journal.pone.0130510

**Published:** 2015-07-01

**Authors:** Mi-Ae Bang, Ji-Hye Seo, Joung-Wook Seo, Gyung Hyun Jo, Seoung Ki Jung, Ri Yu, Dae-Hun Park, Sang-Joon Park

There are errors in the figure captions. Please see the complete, correct captions here.

**Fig 1:**
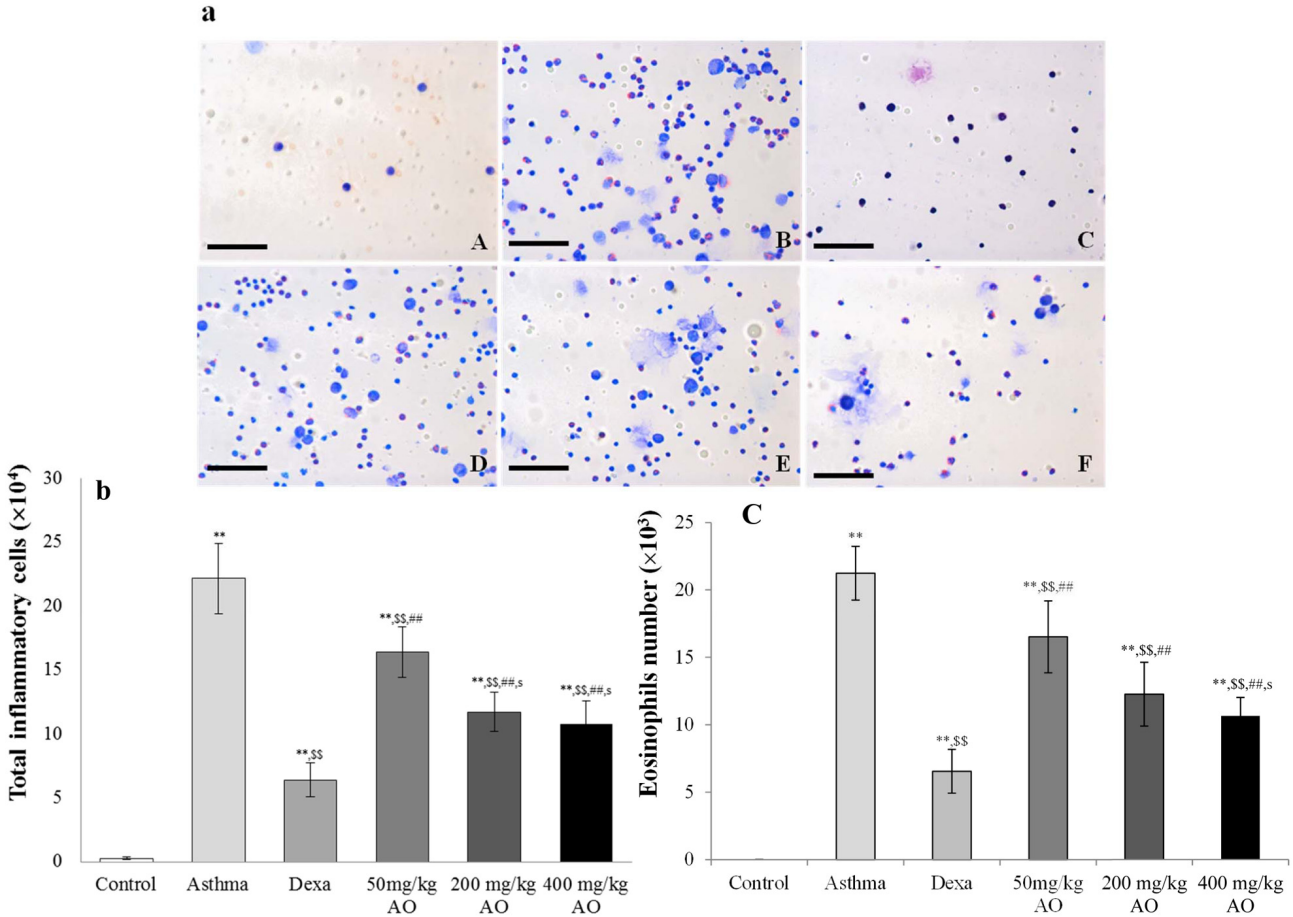
Effects of alginate oligosaccharides (AO) on the ovalbumin (OVA)-induced recruitment of total inflammatory cells and eosinophils in bronchoalveolar lavage fluid (BALF). (a) Total and differential cell counts in BALF were enumerated on slide preparations that were stained with the Kwik-Diff staining set. The numbers of (b) total inflammatory cells and (c) eosinophils in the AO-treated groups were dramatically reduced, compared to the numbers in OVA-challenged, vehicle-treated mice. A, vehicle control; B, asthma induction; C, dexamethasone; D, 50 mg/kg/day AO; E, 200 mg/kg/day AO; F, 400 mg/kg/day AO. Each bar represents the mean ± SEM (n = 6). *p<0.05 vs. control; **p<0.001 vs. control; ^$^ *p*<0.05 vs. asthma induction; ^$$^ *p*<0.001 vs. asthma induction; ^#^ *p*<0.05 vs. dexamethasone; ^##^ *p*<0.001 vs. dexamethasone; ^s^ *p*<0.05 vs. 50 mg/kg/day; ^ss^ *p*<0.001 vs. 50 mg/kg/day; ^p^ *p*<0.05 vs. 200 mg/kg/day; ^pp^ *p*<0.001 vs. 200 mg/kg/day; ^¥^ *p*<0.05 vs. 400 mg/kg/day; ^¥¥^ *p*<0.001 vs. 400 mg/kg/day.Bar = 50 μm.

**Fig 2:**
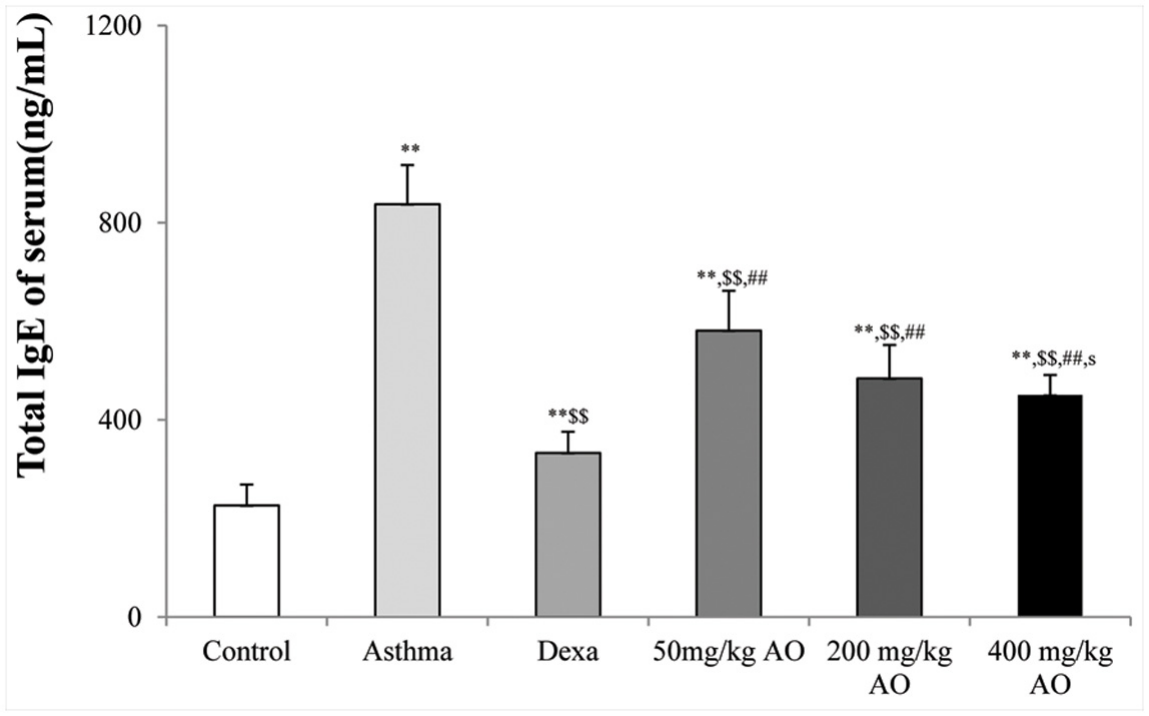
Effects of AO on the OVA-induced upregulation of serum IgE levels. The serum levels of IgE were significantly reduced by AO (*p*<0.01) in a dose-dependent manner, compared with that in OVA-challenged, vehicle-treated mice. A, vehicle control; B, asthma induction; C, dexamethasone; D, 50 mg/kg/day AO; E, 200 mg/kg/day AO; F, 400 mg/kg/day AO. Each bar represents the mean ± SEM (n = 6). *p<0.05 vs. control; **p<0.001 vs. control; ^$^ *p*<0.05 vs. asthma induction; ^$$^ *p*<0.001 vs. asthma induction; ^#^ *p*<0.05 vs. dexamethasone; ^##^ *p*<0.001 vs. dexamethasone; ^s^ *p*<0.05 vs. 50 mg/kg/day; ^ss^ *p*<0.001 vs. 50 mg/kg/day; ^p^ *p*<0.05 vs. 200 mg/kg/day; ^pp^ *p*<0.001 vs. 200 mg/kg/day; ^¥^ *p*<0.05 vs. 400 mg/kg/day; ^¥¥^ *p*<0.001 vs. 400 mg/kg/day.

**Fig 3:**
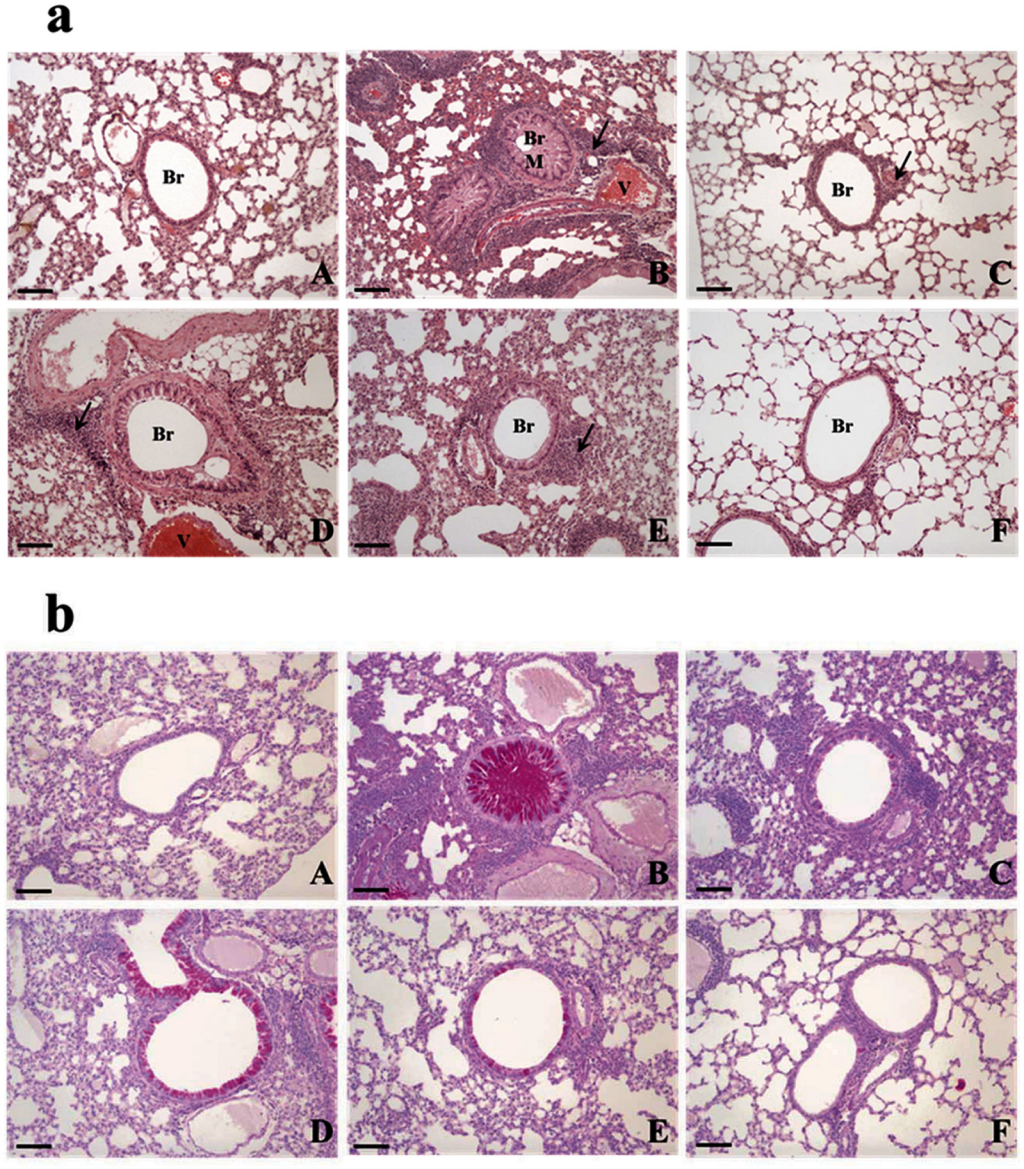
AO dose-dependently suppressed asthma-related histopathological changes in mouse lungs. (a) As observed using the hematoxylin and eosin stain, AO dose-dependently decreased inflammatory cell (eosinophil) infiltration around the vessels and bronchioles, mucus secretion, and goblet cell hyperplasia in the lungs. (b) AO reduced glycoprotein (mucus) secretion in the bronchioles in a dose-dependent manner, as detected by the Periodic acid-Schiff stain. Bar = 10 μm; Arrow = eosinophil infiltration. Br, bronchiole; M, mucus secretion; V, vessel. A, vehicle control; B, asthma induction; C, dexamethasone; D, 50 mg/kg/day AO; E, 200 mg/kg/day AO; F, 400 mg/kg/day AO.

**Fig 4:**
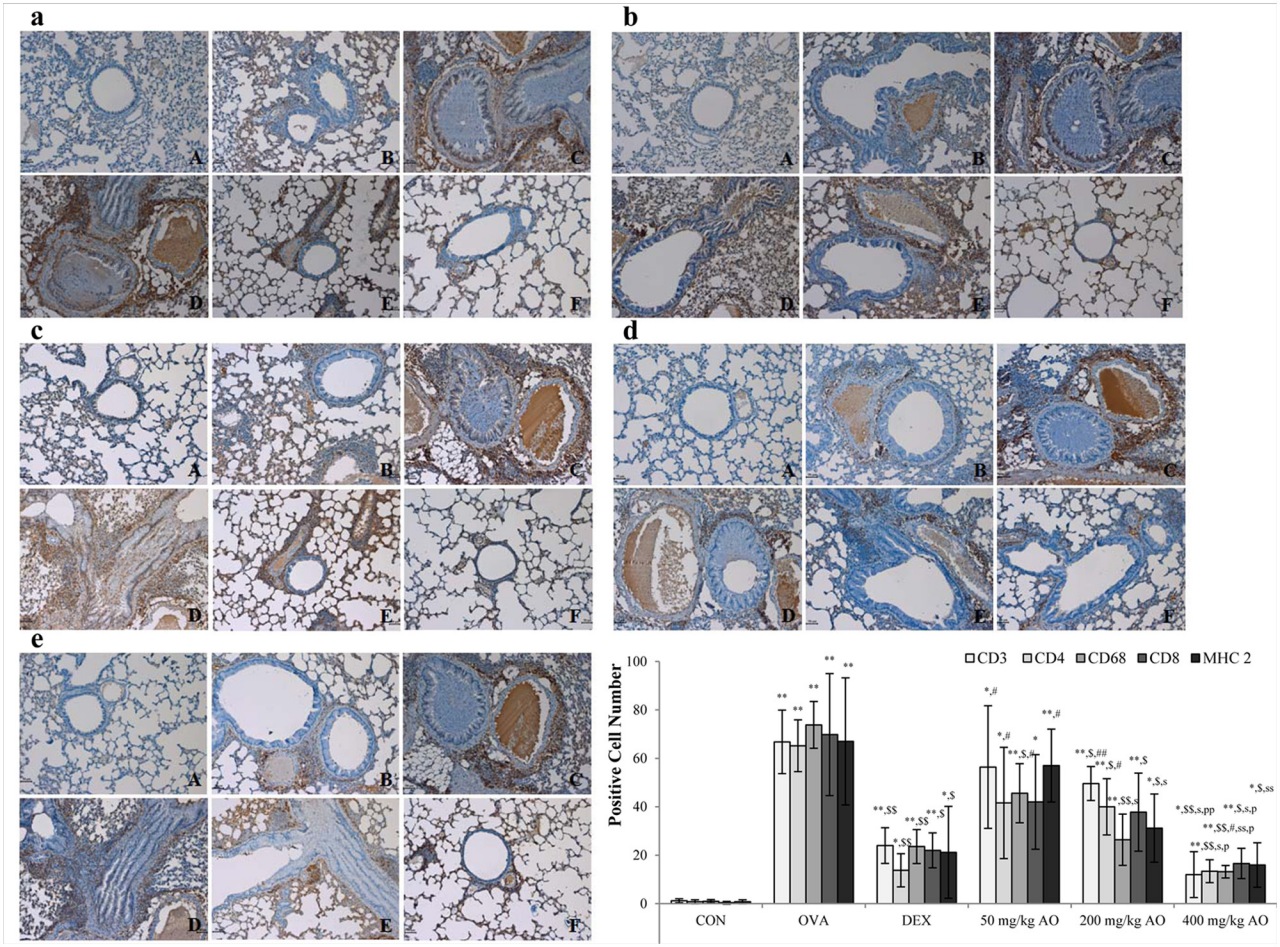
AO dose-dependently suppressed the expression of T-helper (Th) cells, cytotoxic T cells, and the T-cell co-receptor and inhibited the expression of macrophage and MHC class II in asthma. AO (a) inhibited the expression of CD3^+^ T-cell co-receptors in a dose-dependent manner, (b) significantly suppressed the upregulation of CD4^+^ Th cells, (c) downregulated the expression of CD8^+^ cytotoxic T cells, and (d) decreased CD68^+^ macrophasges. (e) AO also inhibited the expression of MHC class II. Immunopositive cells were counted in five randomly selected non-overlapping fields (×200 magnification) of three separately immunostained lung sections per animal. A, vehicle control; B, asthma induction; C, dexamethasone; D, 25 mg/kg/day ACA; E 50 mg/kg/day ACA. CD3: T-cell co-receptor; CD4: Th cell; CD8: cytotoxic T cell; CD68: macrophage; MHC class II: major histocompatibility complex class II molecules. *p<0.05 vs. control; **p<0.001 vs. control; ^$^ *p*<0.05 vs. asthma induction; ^$$^ *p*<0.001 vs. asthma induction; ^#^ *p*<0.05 vs. dexamethasone; ^##^ *p*<0.001 vs. dexamethasone; ^s^ *p*<0.05 vs. 50 mg/kg/day; ^ss^ *p*<0.001 vs. 50 mg/kg/day; ^p^ *p*<0.05 vs. 200 mg/kg/day; ^pp^ *p*<0.001 vs. 200 mg/kg/day; ^¥^ *p*<0.05 vs. 400 mg/kg/day; ^¥¥^ *p*<0.001 vs. 400 mg/kg/day.

**Fig 5:**
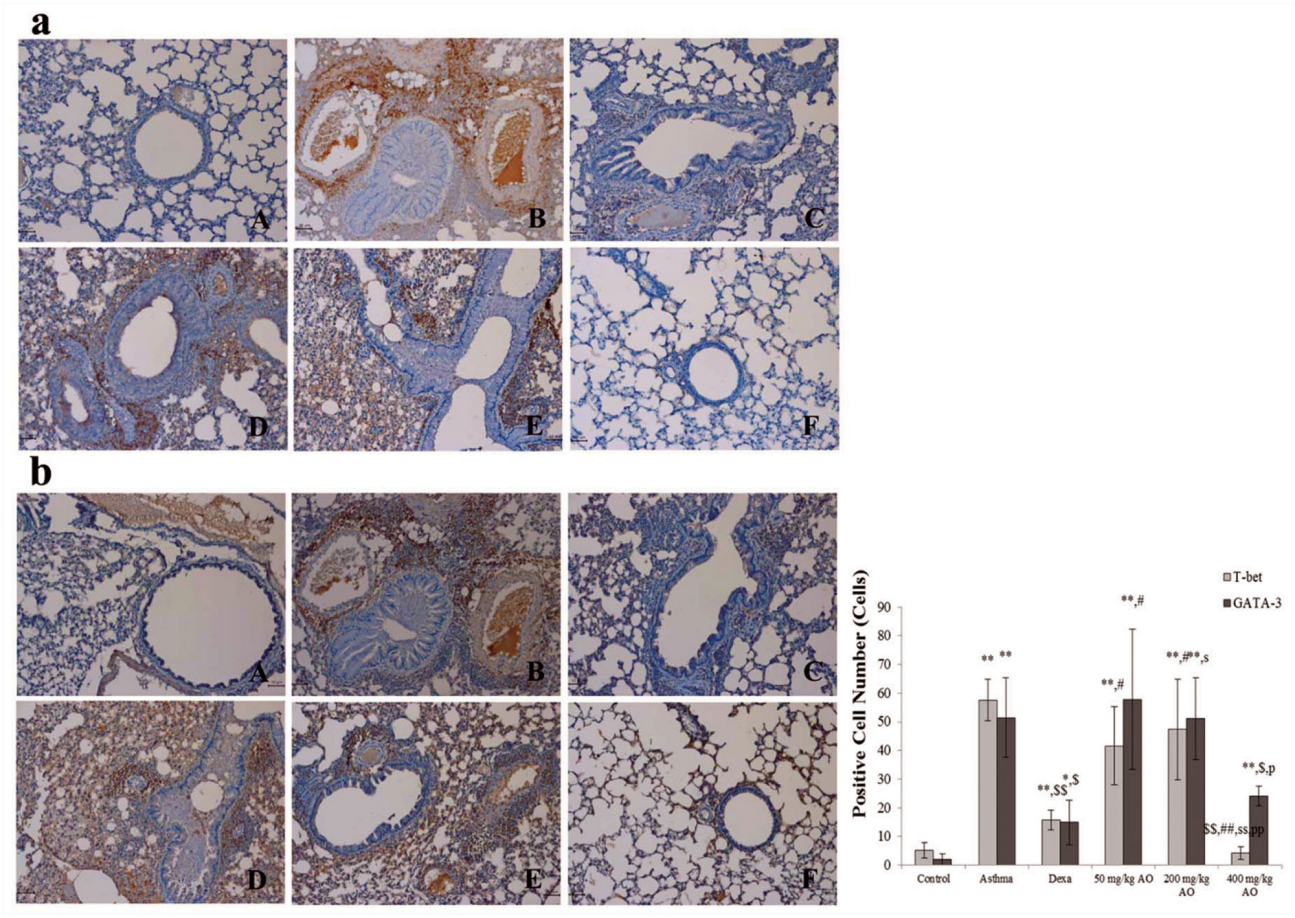
AO reduced the expression of transcription factors to control Th1 cells proliferation and Th2 cells. AO reduced not only the expression of transcription factor, GATA-3 (b), to control Th2 cells proliferation but also the expression of transcription factor, T-bet (a), to do Th1 cells proliferation. Immunopositive cells were counted in five randomly selected non-overlapping fields (×200 magnification) of three separately immunostained lung sections per animal. A, vehicle control; B, asthma induction; C, dexamethasone; D, 50 mg/kg/day AO; E, 200 mg/kg/day AO; F, 400 mg/kg/day. *p<0.05 vs. control; **p<0.001 vs. control; ^$^ *p*<0.05 vs. asthma induction; ^$$^ *p*<0.001 vs. asthma induction; ^#^ *p*<0.05 vs. dexamethasone; ^##^ *p*<0.001 vs. dexamethasone; ^s^ *p*<0.05 vs. 50 mg/kg/day; ^ss^ *p*<0.001 vs. 50 mg/kg/day; ^p^ *p*<0.05 vs. 200 mg/kg/day; ^pp^ *p*<0.001 vs. 200 mg/kg/day; ^¥^ *p*<0.05 vs. 400 mg/kg/day; ^¥¥^ *p*<0.001 vs. 400 mg/kg/day.

**Fig 6:**
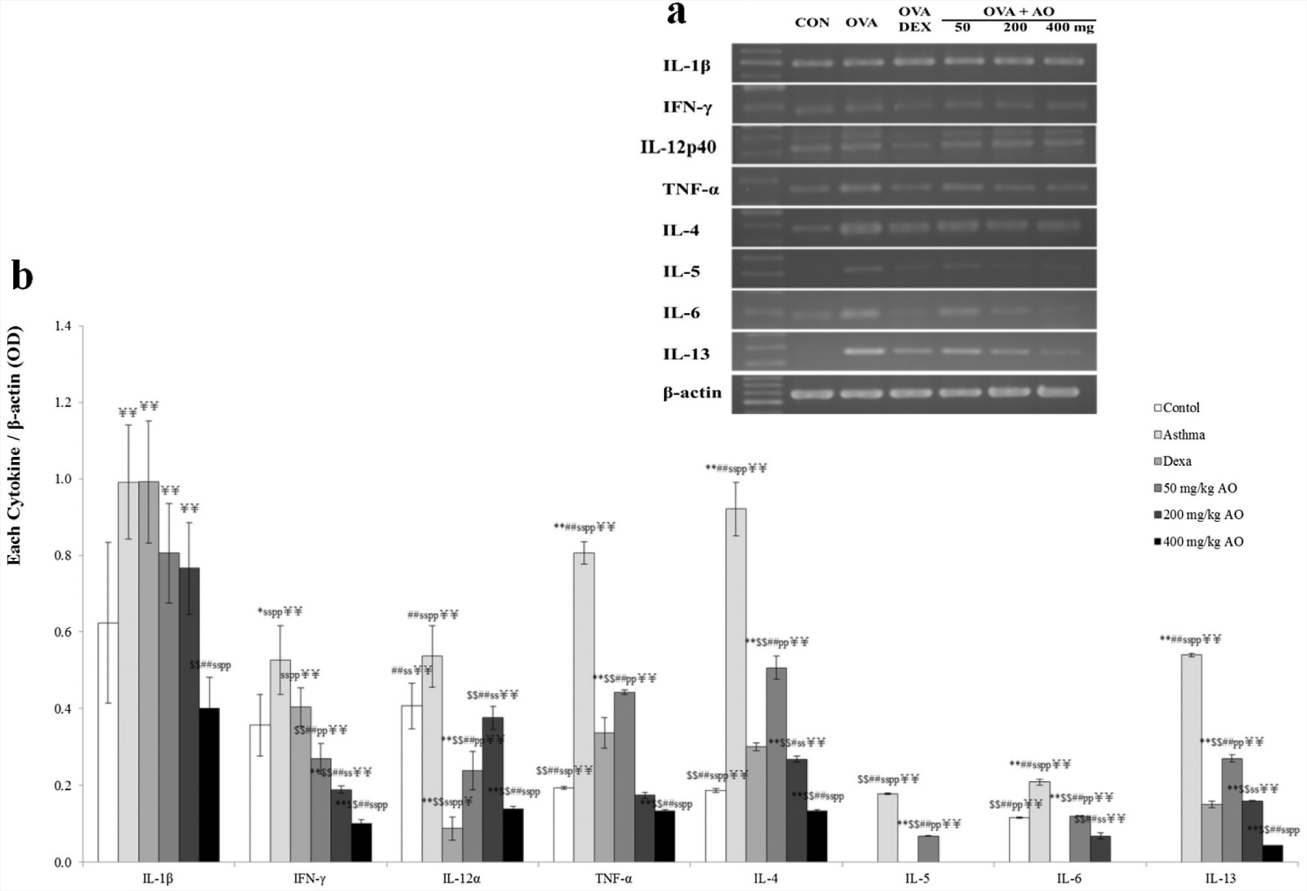
AO suppressed the expression of Th1/2-related cytokines in OVA-induced asthma. Treatment with 400 mg/kg/day AO for 5 days suppressed the expression of *IL-1β* mRNA. AO also decreased the mRNA levels of Th2-related cytokines (TNF-α, IL-4, IL-5, IL-6, and IL-13) and Th1-related cytokines (IFN-) via dose-dependent manners, and it significantly inhibited the expression of *IL-5*, *IL-6*, and *IL-13* mRNA. AO slightly increased the mRNA level of *IL-12α* compared to that of control, and treatment with dexamethasone inhibited IL-12α mRNA expression, which was recovered by treatment with 40 mg/kg/day or 200 mg/kg/day AO. At 400 mg/kg/day, AO down-regulated IL-12α mRNA expression. *p<0.05 vs. control; **p<0.001 vs. control; ^$^ *p*<0.05 vs. asthma induction; ^$$^ *p*<0.001 vs. asthma induction; ^#^ *p*<0.05 vs. dexamethasone; ^##^ *p*<0.001 vs. dexamethasone; ^s^ *p*<0.05 vs. 50 mg/kg/day; ^ss^ *p*<0.001 vs. 50 mg/kg/day; ^p^ *p*<0.05 vs. 200 mg/kg/day; ^pp^ *p*<0.001 vs. 200 mg/kg/day.

**Fig 7:**
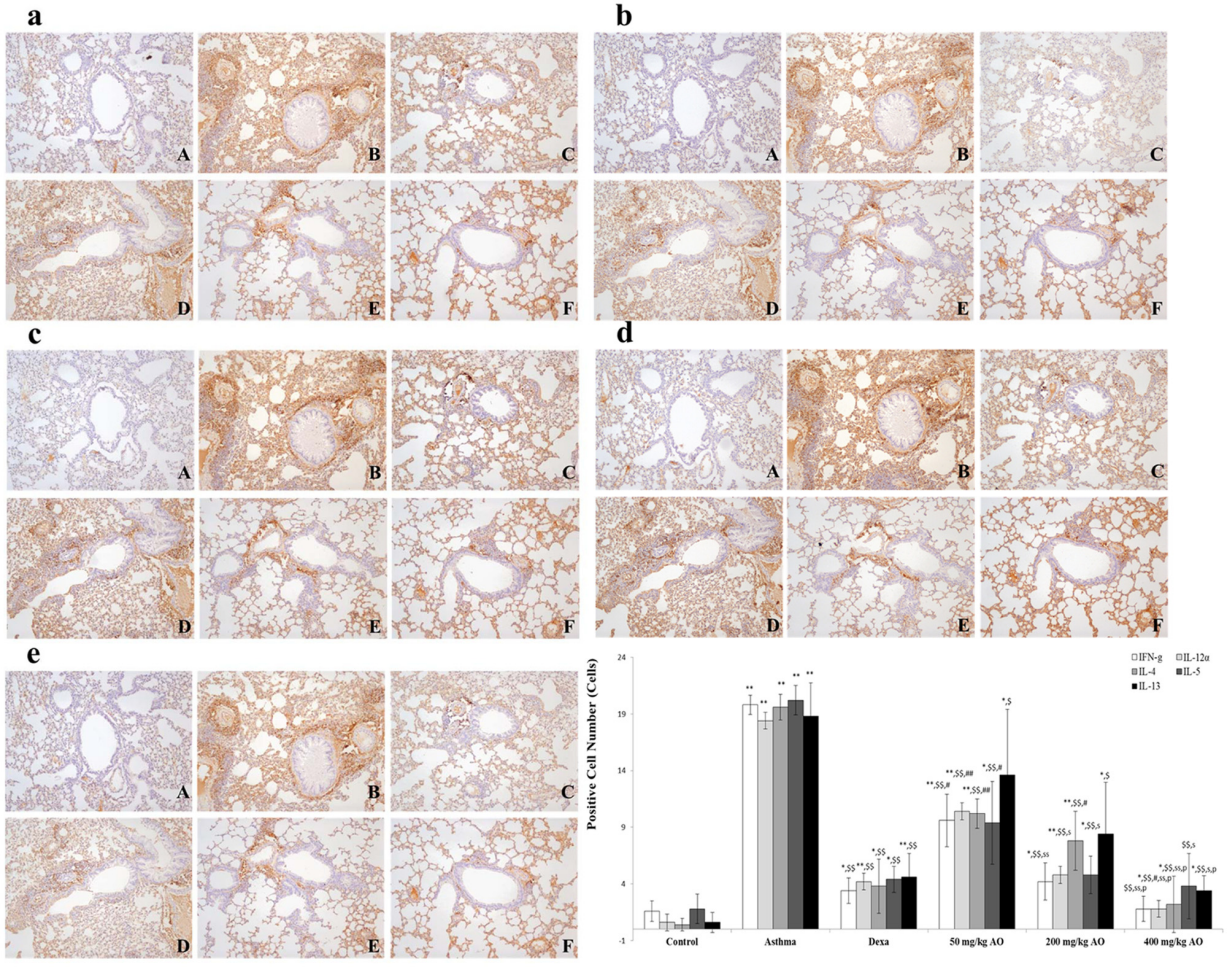
AO reduced the expression of Th1- and Th2-related cytokines. OVA induced the expression of Th1- and Th2-related cytokines. AO reduced the expression of Th1-related cytokines, such as (a) IFN-γ and (b) IL-12α, and Th2-related cytokines, such as (c) IL-4, (d) IL-5, and (e) IL-13, in the lungs. Immunopositive cells were counted in five randomly selected non-overlapping fields (×200 magnification) of three separately immunostained lung sections per animal. A, vehicle control; B, asthma induction; C, dexamethasone; D, 50 mg/kg/day AO; E, 200 mg/kg/day AO; F, 400 mg/kg/day. *p<0.05 vs. control; **p<0.001 vs. control; ^$^ *p*<0.05 vs. asthma induction; ^$$^ *p*<0.001 vs. asthma induction; ^#^ *p*<0.05 vs. dexamethasone; ^##^ *p*<0.001 vs. dexamethasone; ^s^ *p*<0.05 vs. 50 mg/kg/day; ^ss^ *p*<0.001 vs. 50 mg/kg/day; ^p^ *p*<0.05 vs. 200 mg/kg/day; ^pp^ *p*<0.001 vs. 200 mg/kg/day; ^¥^ *p*<0.05 vs. 400 mg/kg/day; ^¥¥^ *p*<0.001 vs. 400 mg/kg/day.

**Fig 8:**
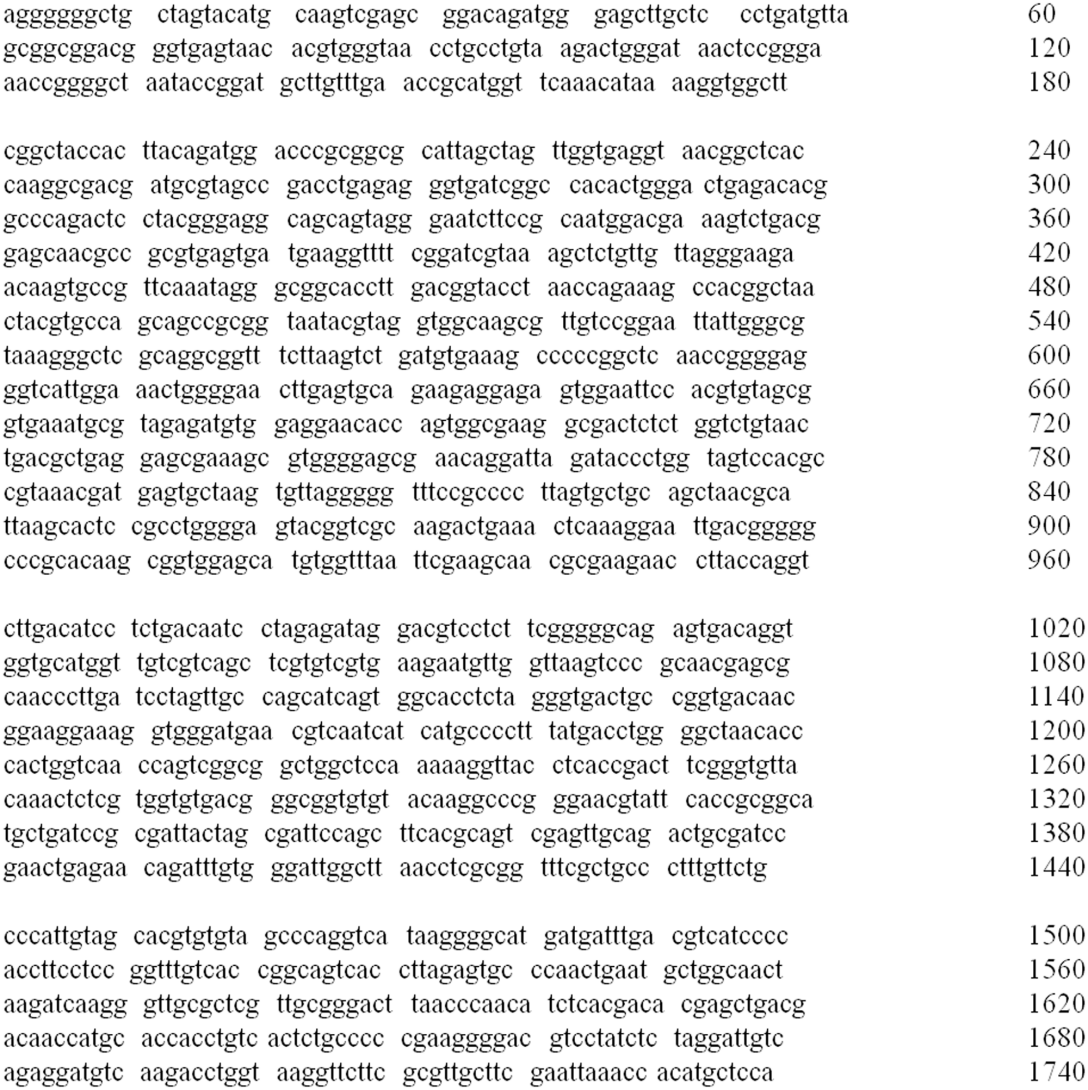
16S rRNA sequences of *Bacillus subtilis* KCTC 11782BP.
