# Supplementary figures and images for: Bacillus subtilis KCTC 11782BP-Produced Alginate Oligosaccharide Effectively Suppresses Asthma via T-Helper Cell Type 2-Related Cytokines
Source: PLoS One. 2015 Feb 6;10(2):e0117524. doi: 10.1371/journal.pone.0117524 (PMC4319839; doi:10.1371/journal.pone.0117524)

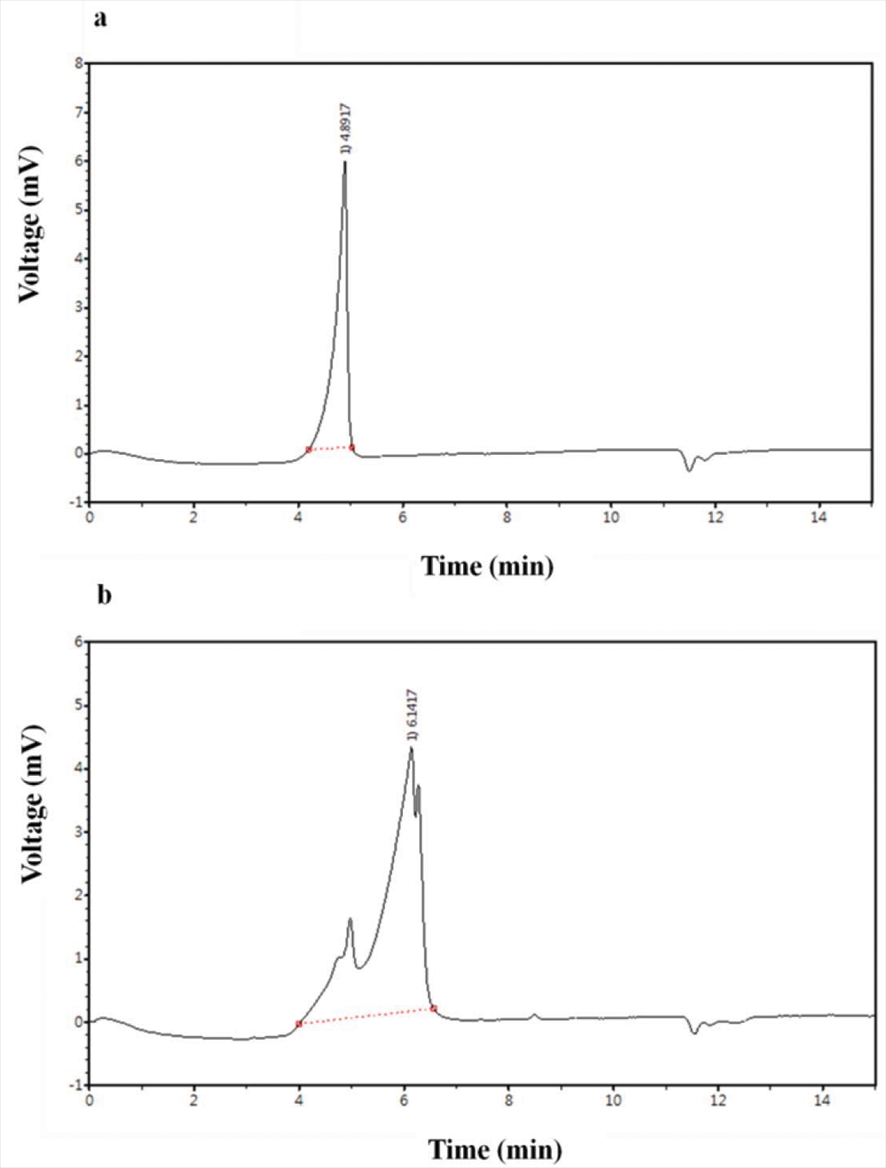

Supplement: S1 Fig — (a) The peak of mannuronate oligosaccharides DP5 was increased for about 1 min after the standard was injected, from the 4-min mark to the 5-min mark. (b) The peak of Bacillus subtilis KCTC 11782BP-produced AO was increased for about 1.5 min after the standard was injected, from the 4-min mark to the 5.5-min mark. (TIF) [file pone.0117524.s001.tif]
